# Prognostic Factors Analysis and Nomogram Construction of Dual Primary Lung Cancer: A Population Study

**DOI:** 10.1155/2020/7206591

**Published:** 2020-02-19

**Authors:** Cong-kuan Song, Zi-xin Guo, Xiao-yan Shen, Yu-jin Wang, Qing-wen Wang, Dong-hu Yu, Chen Chen, Xiao-ping Liu, Jing-yu Huang, Sheng Li, Weidong Hu

**Affiliations:** ^1^Department of Thoracic Surgery, Zhongnan Hospital of Wuhan University, Wuhan 430071, China; ^2^Hubei Key Laboratory of Tumor Biological Behaviors & Hubei Cancer Clinical Study Center, Wuhan 430071, China; ^3^Department of Cardiovascular Surgery, Renmin Hospital of Wuhan University, Wuhan 430071, China; ^4^Department of Biological Repositories, Zhongnan Hospital of Wuhan University, Wuhan 430071, China; ^5^Human Genetics Resource Preservation Center of Hubei Province, Wuhan 430071, China

## Abstract

As a special type of lung cancer, multiple primary lung cancer (MPLC) has unique biological characteristics, and its research remains limited. The aim of our research was to identify prognostic factors and construct a prognostic nomogram of dual primary lung cancer (DPLC). A population cohort study of patients with DPLC was conducted using the extracted data from the Surveillance, Epidemiology, and End Results (SEER) database. Relevant survival variables were identified using the Cox proportional hazard model. Prognostic nomogram was performed and its predictive performance was validated via the modeling and validating cohort data. Additionally, propensity score matching (PSM) was also applied to evaluate whether surgery affected the OS of this study population. 5411 eligible DPLC patients were included in this study cohort, with 41.0% of 3-year OS rate and 27.7% of 5-year OS rate. Age, sex, race, grade, stage, lymph node (LN) metastasis, histological type, primary site, and surgery were considered to be prognostic factors of OS. The C-indexes of the established nomogram were 0.70 (95% CI (0.69, 0.71)) in the modeling group and 0.70 (95% CI (0.68, 0.72)) in the validation group, which showed an ideal model discrimination ability. AUC and calibration plots of 3- and 5-year OS also proved the good performance of the established nomogram. After 1 : 1 PSM, surgery can potentially reduce the risk of OS (HR = 0.63, 95% CI: 0.56–0.72) of DPLC. The prognostic nomogram with reliable performance was developed to predict 3- and 5-year OS rates, which could assist clinicians to make more reasonable survival prediction for DPLC patients. For patients without absolute surgical contraindications, surgery should be actively considered.

## 1. Introduction

Since 1975, when Martini and Melamed proposed the diagnostic criteria [1] for multiple primary lung cancer (MPLC) to distinguish it from intrapulmonary metastasis, the reports of MPLC have been increasing. Despite the continuous improvement of medical technology and systematic treatment of lung cancer, the survival prognosis of patient with MPLC is still not optimistic. Accurate prediction of survival rate of patients with MPLC is of great significance for clinical treatment decisions. Many clinical factors were reported to be related to the survival prognosis of MPLC [2–5], such as age, sex, lymph nodes, tumor stage, and histological type. But so far, there is still no clear conclusion on the prognostic factors of MPLC patients, and few large multicenter studies have evaluated them so as to make reasonable and accurate prediction for the survival prognosis of MPLC patients. Although TNM staging system is the most commonly used method to determine prognosis, it has limitations and the survival time of patients with the same histological type and the same TNM stage still varies greatly. In addition, as a special type of lung cancer, MPLC has special biological characteristics, and the commonly used TNM staging standard is not suitable for the selection of MPLC treatment decisions and prognosis judgment. Therefore, it is required to seek a more refined method to predict the survival of MPLC patients. A nomogram is a good choice for this purpose. In recent years, nomogram has been widely used to evaluate the prognosis of patients with cancer because it can include various prognostic factors, quantify the effects of these factors on survival prognosis, and visualize the results so as to predict the survival rate of patients [6–8]. In this study, we selected dual primary lung cancer (DPLC) patients as the research objects because the vast majority of MPLC was DPLC. We analyzed the patient's available data in the Surveillance, Epidemiology, and End Results (SEER) database. And the Cox proportional hazard model was utilized to identify prognostic factors and develop a prognostic nomogram to establish a relatively systematic evaluation system so as to accurately predict the 3-year and 5-year overall survival (OS) rates of patients with DPLC. Propensity score matching (PSM) was considered more suitable for nonrandomized controlled studies due to its ability of reducing the potential selection bias [9]. We also used the method of PSM to evaluate the impact of surgery on OS in DPLC patients.

## 2. Patients and Methods

### 2.1. Data Source

The SEER database, full name (Surveillance, Epidemiology, and End Results), is the authoritative cancer statistics database in the United States, which records the morbidity, mortality, and disease status of millions of patients with malignant tumors in some states and counties (18 registration sites) in the United States since 1973. We accessed the SEER database from the website (http://seer.cancer.gov/data/). These data from the SEER database are open to the public for research purposes. This study was also approved by the Institutional Research Committee of Zhongnan Hospital of Wuhan University.

### 2.2. Patient Selection

Data from 5411 patients were extracted from the SEER database, who had been diagnosed as DPLC from 2004 to 2015. Patients meeting the following criteria were included in this study: ① year of diagnosis between 2004 and 2015; ② site and morphology (site recoded ICD-O-3/WHO 2008: lung and bronchus); and ③ events (1 of 124 selected for display): lung and bronchus. Furthermore, patients with three or more primary lung cancers were excluded from this study; those without clear status, survival time, AJCC Stage, and AJCC N were also removed, either in the first primary lung cancer (FPLC) or in the second primary lung cancer (SPLC). The variables collected included age at diagnosis, sex, race record, primary site label, laterality, grade, ICO-O-3 Hist/behav, malignancy, the time interval (months since FPLC), AJCC Stage, AJCC N, COD to site recode, Rx Summ-Surg Prim Site (1998+), radiation record, and chemotherapy record. The patients in the study cohort must include complete data in the abovementioned variables. The patient selection process was summarized in [Supplementary-material supplementary-material-1].

### 2.3. Neoplastic Grade and Stage

According to the grades and stages of FPLC and SPLC of the same patient, we took the grade with poorer differentiation and later stage of the two (FPLC and SPLC) as the final grade and stage of this patient. Grades included I, II, III, IV, and unknown, and all patients were staged as IA, IB, IIA, IIB, IIIA, IIIB, and IV in this study.

### 2.4. Statistical Analysis

In our study, OS started from the diagnosis of SPLC. OS rates of all variables were calculated using Kaplan–Meier method by the SPSS version 23.0 (IBM SPSS Inc., Chicago, IL, USA). Simple random sampling was performed with the random sampling function (sample ( ) function) in version 3.6.0 of R software, and patients were randomly divided into the modeling and validation groups by a ratio of 7 to 3, as shown in Table 1. All variables in the modeling group were considered in univariate and multivariate survival analysis by using the Cox proportional hazard model. Additionally, we also utilized the proportional hazards model to estimate OS hazard ratios for prognostic factors, which included age, sex, race, grade, histological type, primary site, interval (months since FPLC), AJCC Stage, AJCC N, surgery, radiation, and chemotherapy. In order to reduce the interference of confounding factors and improve the accuracy of predictive value of the nomogram, univariate survival analysis was performed for all variables, followed by multivariate analysis for statistically significant variables (*p* < 0.05). And then we used all relevant independent prognostic factors of OS to construct their prognostic nomogram at 3- and 5-year OS. The nomogram was developed with “rms” package. The AUC and C-index were applied to evaluate the predictive value of the established nomogram. The value of the C-index statistic ranged from 0.5 to 1.0, and the higher the C-index, the higher the predictive value [10]. Moreover, the performance of the prognostic nomogram was also assessed through internal validation (the modeling and verification groups). Bootstraps with 1000 resamples were adopted to decrease overfit bias. Test level *a* = 0.05. In the ideal calibration curve, the predicted value is equal to the actual observed value, and the curve will be infinitely close to the ideal 45° oblique line. Finally, PSM method was used to minimize the substantial differences that exist in terms of clinical characteristics between the two different groups (no surgery group and surgery group), which can better evaluate the effect of surgery on OS in patients with DPLC. We adopted the R software (version 3.6.0) making use of the “MatchIt” package for calculating propensity scores. A 1 : 1 matched analysis was performed using the nearest neighbor method with a caliper of 0.05.

## 3. Results

### 3.1. Clinical and Pathological Characteristics of All Patients

We identified 5411 DPLC patients diagnosed from 2004 to 2015. Their clinicopathological features of FPLC and SPLC are shown in Table 2. The median age at diagnosis of FPLC was 67 years (range: 25 to 94 years), of which age < 65 accounted for 37.6% and age ≥ 65 was 62.4%. And the median age of SPLC was 71 years (range: 25 to 99 years), of which 25.3% were age < 65 and 74.7% were age ≥ 65. The proportion of men was slightly lower (46.52%) than that of women (53.48%). The largest proportion of these patients was white (85.25%), followed by black (9.76%). The most common histological type in FPLC and SPLC was adenocarcinoma, accounting for 36.72% and 36.46%, respectively. Similarly, Stage IA was the most common in FPLC (36.50%) and SPLC (42.95%). On the basis of combining the same patient's FPLC and SPLC information, we reorganized the final clinicopathological characteristics of all patients. The final age was age at diagnosis of SPLC. The grade with poorer differentiation and later stage of the two were taken as the final grade and stage of this patient, as shown in Table 3. It can be seen from the table that the patients whose FPLC and SPLC were both adenocarcinoma (named “Aden-aden”) had 1000 people, accounting for 18.5% and their 3- and 5-year OS rates were, respectively, 47.4% and 34.9%. The vast majority of patients (65.7%) in this study had two primary tumors (FPLC and SPLC) on different sides, having a 42.0% of 3-year OS rate and 28.4% of 5-year OS rate. Stage IA patients accounted for 16.7% in this study population, who had higher 3- and 5-year OS rates than those in other stages (*p* < 0.001). In addition, there were 1866 patients receiving surgical treatment for both FPLC and SPLC (named “Yes-Yes”) and 1152 patients not (named “No-No”), accounting for 34.5% and 21.3%, respectively. Patients with surgery were associated with higher OS (*p* < 0.001).

### 3.2. Analysis of Factors Influencing Survival and Prognosis

**Figure 1:**
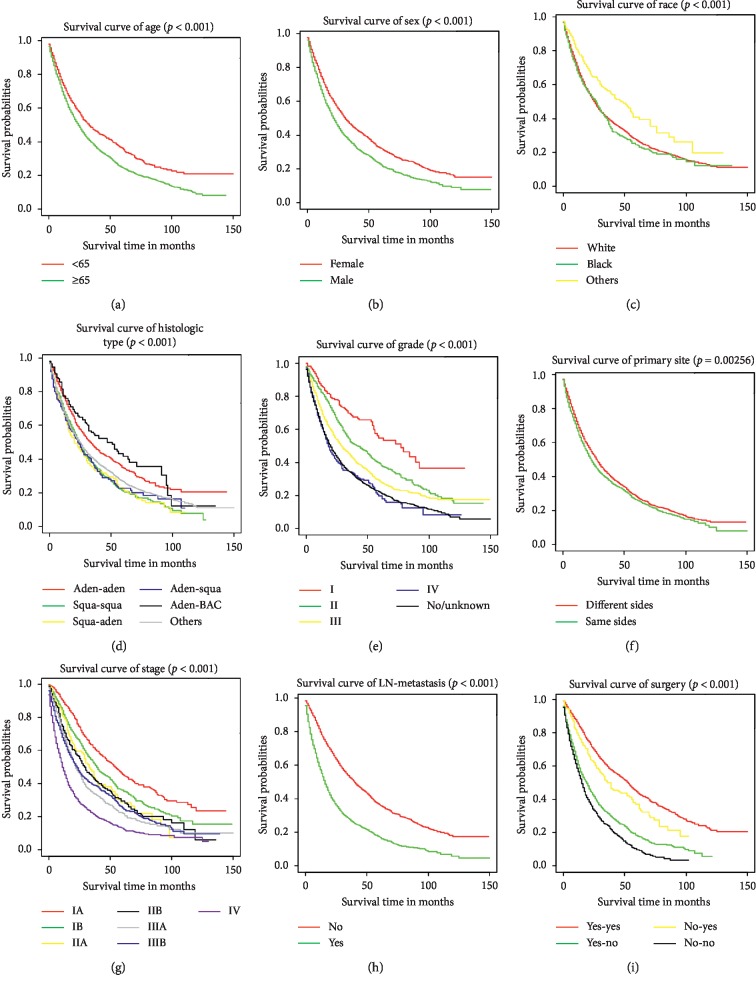
Kaplan–Meier estimated specific survival in patients with DPLC stratified by age (a), sex (b), race (c), histological type (d), grade (e), primary site (f), stage (g), LN metastasis (h), and surgery (i).

Univariate and multivariate Cox analysis was performed on 3791 patients in the modeling group and the results showed that age at diagnosis, gender, race, neoplastic grade, stage, LN metastasis, histological type, location, and surgery were closely related to survival prognosis of DPLC patients (*p* < 0.05), as shown in Table 4. In order to more vividly reflect the relationship between independent risk factors and survival time, 5411 DPLC patients were analyzed and survival curves were drawn using the Kaplan–Meier method (Figure 1). It can be seen from Table 4 and Figure 1 that these patients who had older age, worse grade, and later stage (except IIIA and IIIB) were linked to worse prognosis. The prognosis of black and white race was not significantly abnormal, and the prognosis of the other race was better than that of white race (*p* < 0.001). In addition, the prognosis of male patients was worse than that of female patients (*p* < 0.001), and the prognosis of patients with lymph node metastasis was worse than that of patients without metastasis (*p* < 0.001). Compared to patients with squamous cell carcinoma in both FPLC and SPLC, patients with adenocarcinomas in both FPLC and SPLC had better prognosis (*p*=0.002). The survival prognosis of patients who did not receive surgical treatment for either FPLC or SPLC was worse than that of patients who received surgical treatment whether FPLC or SPLC (*p* < 0.001).

### 3.3. The Predictive Effect of Nomogram on Overall Survival

**Figure 2:**
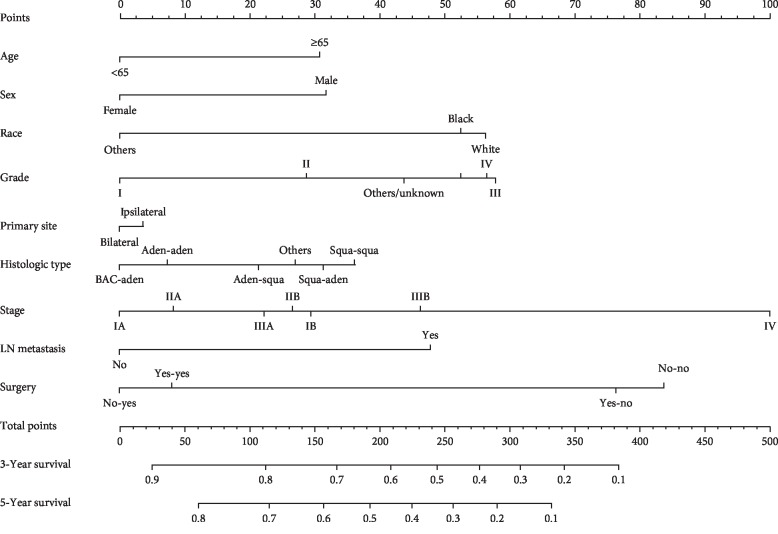
Prognostic nomogram of overall survival in DPLC patients. Nomogram to predict 3- and 5-year OS rates of DPLC patients. The factors of age, sex, race, grade, primary site, histologic type, stage, LN metastasis and surgery were included in the model. Histological type: “Aden-aden” = both first and second primary lung cancer are adenocarcinomas; “Squa-squa” = both first and second primary lung cancer are squamous cell carcinomas; “Squa-aden” = FPLC is squamous cell carcinoma and SPLC is adenocarcinoma; “Aden-squa” = FPLC is adenocarcinoma and SPLC is squamous cell carcinoma; “BAC-aden” = FPLC is bronchioloalveolar cancer and SPLC is adenocarcinoma; Surgery: “Yes-yes” = patients received corresponding treatment for both first and second primary lung cancer; “Yes-no” = patients received corresponding treatment for FPLC and not for SPLC; “No-yes” = patients received corresponding treatment for SPLC and not for FPLC; “No-no” = patients did not received corresponding treatment for first and second primary lung cancer.

**Figure 3:**
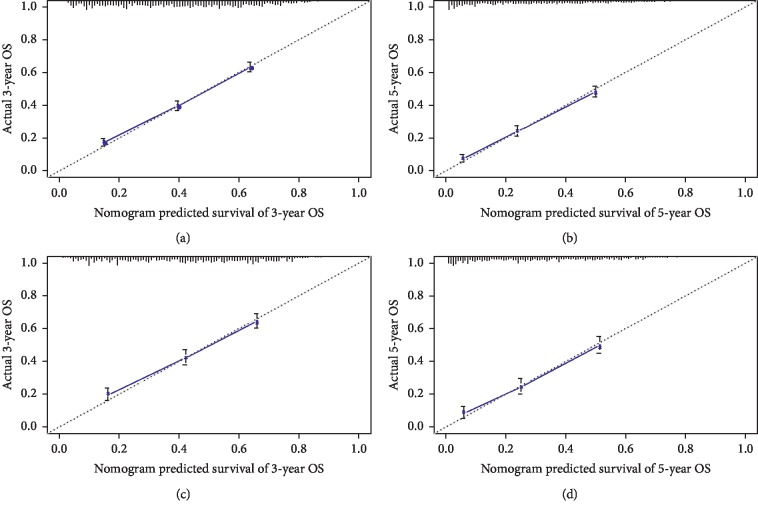
The calibration curves for predictions of 3-year and 5-year overall survival in the modeling (a, b) and validation (c, d) groups.

Nomogram included all statistically significant prognostic factors in the Cox proportional hazard model, including age, sex, race, neoplastic grade, histological type, primary site, stage, LN metastasis, and surgery. The prediction results of 3- and 5-year OS rates are shown in Figure 2. According to the different classification of each feature, points are projected upward to get the score of each item. The total points are calculated by adding all the points, and the survival rate of patients can be calculated by projecting the total points downward. The higher the score, the worse the survival prognosis. This nomogram can be used to predict the survival rate of different patients according to their own conditions, so as to improve the efficiency and accuracy of the prediction. In this study, the established nomogram was verified with bootstrap method. The number of self-sampling B was 1000, and the validation results showed that the C-indexes of the modeling and validation groups were 0.70 (95% CI (0.69, 0.71)) and 0.70 (95% CI (0.68, 0.72)), respectively, both of which had good predictive value. The ROC curves and AUC also proved this conclusion ([Supplementary-material supplementary-material-1] and [Supplementary-material supplementary-material-1]). Additionally, the calibration curves of 3- and 5-year OS rates of the modeling and validation groups are shown in Figure 3, from which we could see that the calibration curves of both modeling and validation groups were close to the ideal 45° dotted line, indicating a good consistency between the predicted value and the actual observed value.

### 3.4. Propensity Score Matching of Surgery in This Study Population

In this study, surgery was regarded as the prognosis factor of OS and the survival prognosis of patients who did not receive surgical treatment for either FPLC or SPLC was worse than that of patients who received surgical treatment in whether FPLC or SPLC (Table 4 and Figure 1). However, there were significant differences in some variables between the patients with surgery and the patients without surgery in the study cohort, including race, histological type, interval, grade, stage, LN metastasis, radiation, and chemotherapy ([Supplementary-material supplementary-material-1]). So PSM method was used to reduce the differences in these variables between the two groups so as to better evaluate the effect of surgery. The matching effect of the method can be seen from [Supplementary-material supplementary-material-1] and [Supplementary-material supplementary-material-1]. We found 666 paired DPLC patients with nearly balanced variables after 1 : 1 PSM ([Supplementary-material supplementary-material-1]).

**Figure 4:**
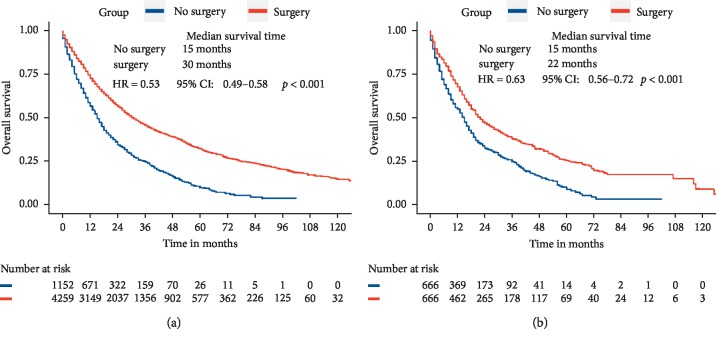
Overall survival in DPLC patients with or without surgery before (a) and after (b) 1 : 1 propensity score matching.

Before PSM, median survival time of the patients who received surgical treatment was significantly longer than that without surgery (30 months vs. 15 months), which was consistent with the results after PSM (22 months vs. 15 months). In addition, before PSM, the 3-year OS in the surgery and no surgery groups was 45.3% and 24.3%, respectively (*p* < 0.001). And after PSM, there was 37.7% of 3-year OS rate in the surgery group while there was 24.5% of 3-year OS rate in the no surgery group (*p* < 0.001). Surgery seemed to be related to the low risk of OS of DPLC before (HR = 0.53 95% CI: 0.49–0.58) and after (HR = 0.63 95% CI: 0.56–0.72) PSM (Figure 4).

## 4. Discussion

With the rapid development of medical technology, the improvement of people's living standards, and the extension of survival of lung cancer patients, the detection rate of MPLC continues to improve. Thakur and his colleagues observed that SPLC occurred in 3% of 156,494 patients with primary lung cancers, and the incidence of SPLC was 1.10% per year. The risk did not stabilize over time. The study also found that patients with a history of lung cancer had a higher risk of developing new primary lung cancer than the general population [11]. In recent years, more and more attention has been paid to the survival prognosis of MPLC. However, there were no large studies to evaluate prognostic factors and construct a prognostic nomogram of DPLC. In our study, we found that age at diagnosis, gender, race, neoplastic grade, stage, LN metastasis, histological type, tumor location, and surgery were closely associated with OS of DPLC through the univariate and multivariate Cox regression analysis, as shown in Table 4 and Figure 1. All DPLC patients were involved in the study cohort, with 41.0% of 3-year OS rate and 27.7% of 5-year OS rate. The survival rate was lower than in other studies [12–14]. There were several reasons for this difference. Firstly, the cases included in these studies were all surgically treated patients, and surgery can significantly improve the survival rate of DPLC patients, as our study and others concluded [15–18]. Secondly, the starting point for calculating survival time in these studies was different and the survival time was measured from the diagnosis of SPLC in our study. Thirdly, these studies were small sample and single center retrospective studies with obvious selective bias.

It is worth noting that there are still no clear treatment guidelines and plans for MPLC. At present, it is generally agreed that surgical treatment is the first choice for MPLC, and other treatment methods can be combined for lesions that cannot be completely resected. In order to reduce the influence of hybrid factors, the Cox proportional hazard model and PSM method were used to evaluate the impact of surgery on DPLC patients' survival, and the results showed that surgery can improve the long-term survival of DPLC patients.

Furthermore, in our study, age, sex, race, LN metastasis, stage, and neoplastic grade were also regarded as independent prognostic factors for OS of DPLC patients; the same results were also observed in other studies [3–5, 15]. Tanvetyanon and his colleagues reported that adenocarcinoma was related to better outcomes [2], which was consistent with our research results. Compared to patients with other histological types, patients with “BAC-aden” or with “aden-aden” had better prognosis (*p* < 0.001). In addition, tumor location was considered to be associated with prognosis in DPLC patients in the univariate survival analysis; that is, the prognosis was better on the opposite side than on the same side. A similar result has been previously reported [2]. However, this conclusion was contrary to that of Ishikawa and his colleagues [19]. In multivariate survival analyses, tumor location was not statistically significant (*p*=0.479) in our study, which was similar to the results of others [14, 20].

Whether the time interval between FPLC and SPLC is related to OS of the DPLC patients has been controversial. Some studies suggested that the longer the interval, the better the prognosis [21–24]. Aziz and his colleagues argued that the longer interval was associated with less invasive SPLC [24]. However, other studies had not come to the same conclusion [25, 26]. Some of these studies reported that the prognosis of synchronous MPLC was better than that of metachronous MPLC, while a meta-analysis suggested that time interval had nothing to do with OS of MPLC patients [27]. In our study, the relationship between time interval and prognosis of DPLC patients was reversed in univariate and multivariate survival analyses. And the reason for our results was that the SEER database can only provide a limited number of fields so that there were many other unknowns that cannot be included in the analysis and thus the interference of confounding factors cannot be completely eliminated. That is also why we did not treat the time interval as an independent prognostic factor for DPLC patients. In addition, a lot of researches have demonstrated the benefits of chemotherapy and radiotherapy in MPLC patients [14, 28–30], and it has been agreed that chemotherapy and radiotherapy can improve the survival of MPLC patients. However, our study found that radiotherapy and chemotherapy did not increase the survival advantage of patients (in the univariate survival analysis), so we did not include radiotherapy and chemotherapy into the multivariate survival analysis. Moreover, the SEER database cannot provide the specific chemotherapy plan and time of DPLC patients; some patients were recommended by doctors to receive radiotherapy while the patients gave up radiotherapy and specific radiation information was not available from SEER database. All mentioned above were closely related to the prognosis of them. For these reasons, we did not consider chemotherapy and radiotherapy as predictors of DPLC patients.

Our study has the following advantages. Firstly, our study is the first attempt to use nomogram to predict survival and prognosis of DPLC, including 5411 patients from SEER database in the study cohort. In recent years, some nomograms based on SEER database have been widely used in many studies on a variety of cancers [6, 7, 31, 32]. The SEER database collects a lot of information on the population of 18 registration stations distributed throughout the United States, which accounts for about 28 percent of the US population, with a data accuracy up to 95% [33]. Therefore, it can provide good data support for the construction of clinical prediction models, which is not possible in general single center studies and small sample studies. Secondly, in this study, all prognostic factors mentioned above were included, and different sets of each indicator were quantified to construct a relatively systematic and complete evaluation system. The nomogram based on the above factors had a C-index of 0.70 (95% CI (0.69, 0.71)) in the modeling group and 0.70 (95% CI (0.68, 0.72)) in the verification group, respectively. And the calibration curves of the two groups also showed good consistency, as shown in Figure 3, all of which revealed that the clinical predictive model had relatively ideal predictive value. Therefore, we constructed the prognostic predictive model with good performance, which can assist doctors to evaluate the prognosis of DPLC patients so as to take corresponding measures.

Certainly, our research also has some shortcomings. The first disadvantage is that we considered the clinicopathological characteristics of FPLC and SPLC at the same time when studying the prognosis factors of DPLC patients in order to make the information of DPLC patients more comprehensive and the study more convincing, which made it more difficult to group each individual prognostic factor precisely, such as the specific type of surgery, lymph node status, tumor location, and size for each primary lung lesion. Second, the SEER database does not provide specific chemotherapy regimens of FPLC and SPLC, which can affect the effectiveness of treatment and is closely related to survival. Third, the database also lacks important information such as family history of lung cancer and smoking, which may be the prognostic factors of DPLC. In addition, our study is a retrospective analysis and patients with incomplete information were removed from the study, which inevitably led to selective bias. Considering the shortcomings of retrospective analysis, further prospective analysis should be recommended for prognostic factor assessment.

## 5. Conclusion

In summary, patients with DPLC have poor prognosis with approximately 42.0% of 3-year OS rate and 27.7% of 5-year OS rate. Age at diagnosis, gender, race, neoplastic grade, stage, LN metastasis, histological type, tumor location, and surgery were seen as prognostic factors of OS in DPLC patients. The nomogram based on these factors has good predictive value. Surgical resection is effective treatment for patients with DPLC. Thus, for patients without absolute surgical contraindications, surgery should be actively considered.

## Figures and Tables

**Table 1:** Demograghic and clinicopathological characteristics of the modeling and validation groups.

Variables	Modeling group (*n* = 3791)	Validation group (*n* = 1620)
	No. of patients (%)	No. of patient (%)
*Age*		
<65	959 (25.2)	411 (25.3)
≥65	2832 (74.8)	1209 (74.7)

*Gender*		
Female	2021 (53.3)	873 (53.8)
Male	1770 (46.7)	747 (46.2)

*Race*		
White	3222 (84.9)	1391 (85.8)
Black	385 (10.2)	143 (8.9)
Others	184 (4.9)	86 (5.3)

*Histological type*		
Aden-aden	708 (18.6)	292 (18.1)
Squa-squa	386 (10.1)	192 (11.8)
Squa-aden	176 (4.7)	78 (4.8)
Aden-squa	153 (4.1)	71 (4.4)
BAC-aden	107 (2.8)	40 (2.5)
Others	2261 (59.7)	947 (58.4)

*Interval*		
<6 months	437 (11.5)	180 (11.1)
≥6 months	3354 (88.5)	1440 (88.9)

*Primary site*		
Bilateral	2494 (65.7)	1065 (65.7)
Ipsilateral	1297 (34.3)	555 (34.3)

*Grade*		
I/well	128 (3.4)	52 (3.2)
II/moderate	616 (16.2)	278 (17.2)
III/poor	971 (25.6)	430 (26.6)
IV/undifferentiated	187 (4.9)	67 (4.1)
Others/unknown	1889 (49.9)	793 (48.9)

*Stage*		
IA	646 (17.0)	262 (16.2)
IB	674 (17.8)	324 (20.0)
IIA	95 (2.6)	51 (3.1)
IIB	206 (5.5)	92 (5.6)
IIIA	476 (12.5)	205 (12.7)
IIIB	702 (18.5)	290 (17.9)
IV	992 (26.1)	396 (24.5)

*LN metastasis*		
No/unknown	1990 (52.4)	877 (54.1)
Yes	1801 (47.6)	743 (45.9)

*Surgery*		
Yes-yes	1285 (34.0)	581 (35.8)
Yes-no	1514 (39.9)	616 (38.1)
No-yes	185 (4.8)	78 (4.8)
No-no	807 (21.3)	345 (21.3)

*Radiation*		
Yes-yes	531 (14.1)	214 (13.3)
Yes-no	516 (13.6)	225 (13.8)
No-yes	970 (25.6)	420 (26.0)
No-no	1774 (46.7)	761 (46.9)

*Chemotherapy*		
Yes-yes	520 (13.7)	220 (13.6)
Yes-no	790 (20.8)	359 (22.2)
No-yes	637 (16.9)	278 (17.1)
No-no	1844 (48.6)	762 (47.1)

**Table 2:** The clinicopathological characteristics of FPLC and SPLC in all patients.

Variables	FPLC	SPLC
	No. of patients (%)	No. of patients (%)
*Age*		
<65	2033 (37.57%)	1370 (25.32%)
≥65	3378 (62.43%)	4041 (74.68%)

*Gender*		
Female	2894 (53.48%)	2894 (53.48%)
Male	2517 (46.52%)	2517 (46.52%)

*Race*		
White	4613 (85.25%)	4613 (85.25%)
Black	528 (9.76%)	528 (9.76%)
Others	270 (4.99%)	270 (4.99%)

*Histologic type*		
Adenocarcinoma	1987 (36.72%)	1973 (36.46%)
Squamous cell carcinoma	1386 (25.61%)	1172 (21.66%)
Small cell carcinoma	286 (5.29%)	411 (7.60%)
Bronchioloalveolar cancer	307 (5.67%)	214 (3.95%)
Others	1445 (26.70%)	1641 (30.33%)

*Primary site*		
Left lower lobe	746 (13.79%)	871 (16.10%)
Left upper lobe	1577 (29.14%)	1340 (24.76%)
Right upper lobe	1725 (31.88%)	1479 (27.33%)
Right middle lobe	263 (4.86%)	294 (5.43%)
Right lower lobe	832 (15.38%)	956 (17.67%)
Left main bronchus	38 (0.70%)	82 (1.52%)
Left Lung, Nos	45 (0.83%)	103 (1.90%)
Left overlapping lesion of lung	16 (0.30%)	11 (0.20%)
Right main bronchus	47 (0.87%)	95 (1.76%)
Right Lung, Nos	87 (1.61%)	159 (2.94%)
Right overlapping lesion of lung	35 (0.65%)	21 (0.39%)

*Grade*		
I	603 (11.14%)	591 (10.92%)
II	1869 (34.54%)	1290 (23.84%)
III	1689 (31.21%)	1169 (21.60%)
IV	167 (3.09%)	126 (2.33%)
No/unknown	1083 (20.01%)	2235 (41.30%)

*AJCC stage*		
IA	1975 (36.50%)	2324 (42.95%)
IB	1362 (25.17%)	642 (11.86%)
IIA	128 (2.37%)	127 (2.35%)
IIB	331 (6.12%)	154 (2.85%)
IIIA	561 (10.37%)	427 (7.89%)
IIIB	595 (11.00%)	689 (12.73%)
IV	459 (8.48%)	1048 (19.37%)

*AJCC N*		
N0	3957 (73.13%)	3840 (70.97%)
N1	458 (8.46%)	353 (6.52%)
N2	817 (15.10%)	888 (16.41%)
N3	145 (2.68%)	247 (4.56%)
Nx	34 (0.63%)	83 (1.53%)

*Surgery*		
Yes	1415 (26.15%)	3308 (61.13%)
No	3996 (73.85%)	2103 (38.87%)

*Radiation*		
Yes	1447 (26.74%)	2135 (39.54%)
No/unknown	3925 (73.26%)	3276 (60.46%)

*Chemotherapy*		
Yes	1889 (34.91%)	1655 (30.59%)
No/unknown	3522 (65.09%)	3756 (69.41%)

**Table 3:** The final clinicopathological characteristics of all DPLC patients.

Variables	No. of patients (%)	36-month OS (%)	60-month OS (%)	*P* value
*Age*				<0.001
<65	1370 (25.3)	47.4	36.3	
≥65	4041 (74.7)	38.8	24.7	

*Gender*				<0.001
Female	2894 (53.4)	45.9	32.2	
Male	2517 (46.6)	35.4	22.5	

*Race*				<0.001
White	4613 (85.3)	40.3	27.2	
Black	528 (9.8)	38.9	24.1	
Others	270 (4.9)	55.7	39.5	

*Histologic type*				<0.001
Aden-aden	1000 (18.5)	47.4	34.9	
Squa-squa	578 (10.7)	36.7	21.0	
Squa-aden	254 (4.6)	33.8	22.0	
Aden-squa	224 (4.2)	36.3	21.5	
BAC-aden	147 (2.7)	54.8	41.7	
Others	3208 (59.3)	40.0	26.8	

*Interval*				<0.001
<6 months	437 (11.5)	48.0	35.2	
≥6 months	3354 (88.5)	40.0	26.0	

*Primary site*				0.003
Bilateral	3559 (65.7)	42.0	28.4	
Ipsilateral	1852 (34.3)	38.9	26.4	

*Grade*				<0.001
I/well	180 (3.4)	69.0	53.2	
II/moderate	894 (16.6)	52.3	39.4	
III/poor	1401 (25.8)	44.2	28.6	
IV/undifferentiated	254 (4.6)	34.0	19.8	
Others/unknown	2682 (49.6)	34.1	22.0	

*Stage*				<0.001
IA	908 (16.7)	61.7	45.7	
IB	998 (18.5)	52.9	36.0	
IIA	146 (2.6)	43.5	28.7	
IIB	298 (5.5)	43.7	29.6	
IIIA	681 (12.6)	34.3	22.3	
IIIB	992 (18.4)	39.2	25.4	
IV	1399 (25.7)	21.9	13.1	

*LN metastasis*				<0.001
No/unknown	2867 (52.9)	52.7	36.8	
Yes	2544 (47.1)	27.7	17.3	

*Surgery*				<0.001
Yes-yes	1866 (34.5)	59.6	44.7	
Yes-no	2130 (39.4)	31.7	18.3	
No-yes	263 (4.8)	49.8	34.4	
No-no	1152 (21.3)	24.3	9.6	

*Radiation*				<0.001
Yes-yes	745 (13.8)	33.2	16.9	
Yes-no	741 (13.7)	30.1	18.3	
No-yes	1390 (25.7)	39.5	24.0	
No-no	2535 (46.8)	46.8	34.1	

*Chemotherapy*				<0.001
Yes-yes	740 (13.6)	28.4	17.6	
Yes-no	1149 (21.3)	46.1	32.3	
No-yes	915 (16.9)	29.8	19.6	
No-no	2607 (48.2)	46.1	31.2	

**Table 4:** The prognostic factors for overall survival according to cox proportional hazards regression model.

Variables	No of patients (%)	Univariate cox analysis			Multivariate cox analysis		
		HR	95% CI	*P* value	HR	95% CI	*P* value
*Age*							
<65	959 (25.2)	—	—	—	—	—	—
≥65	2832 (74.8)	1.34	1.22–1.47	<0.001	1.30	1.18–1.43	<0.001

*Gender*							
Female	2021 (53.3)	—	—	—	—	—	—
Male	1770 (46.7)	1.32	1.22–1.42	<0.001	1.31	1.21–1.42	<0.001

*Race*							
White	3222 (84.9)	—	—	—	—	—	—
Black	385 (10.2)	1.02	0.90–1.16	0.76	0.97	0.85–1.10	0.624
Others	184 (4.9)	0.65	0.53–0.80	<0.001	0.62	0.50–0.76	<0.001

*Interval*							
<6 months	437 (11.5)	—	—	—	—	—	—
≥6 months	3354 (88.5)	1.26	1.12–1.43	<0.001	0.98	0.86–1.11	0.745

*Primary site*							
Bilateral	2494 (65.7)	—	—	—	—	—	—
Ipsilateral	1297 (34.3)	1.11	1.02–1.20	0.01	1.03	0.95–1.12	0.474

*Histologic type*							
Aden-aden	708 (18.6)	—	—	—	—	—	—
Squa-squa	386 (10.1)	1.43	1.25–1.66	<0.001	1.28	1.10–1.51	0.002
Squa-aden	176 (4.7)	1.45	1.19–1.78	<0.001	1.23	1.00–1.51	0.046
Aden-squa	153 (4.1)	1.19	0.96–1.48	0.11	1.13	0.90–1.41	0.282
BAC-aden	107 (2.8)	0.86	0.66–1.14	0.29	0.94	0.71–1.24	0.658
Others	2261 (59.7)	1.28	1.15–1.43	<0.001	1.18	1.06–1.33	0.003

*Grade*							
I/well	128 (3.4)	—	—	—	—	—	—
II/moderate	616 (16.2)	1.43	1.06–1.91	0.02	1.28	0.95–1.72	0.101
III/poor	971 (25.6)	2.10	1.58–2.78	<0.001	1.64	1.23–2.19	<0.001
IV/undifferentiated	187 (4.9)	2.50	1.82–3.44	<0.001	1.63	1.18–2.25	0.003
Others/unknown	1889 (49.9)	2.71	2.05–3.57	<0.001	1.46	1.10–1.94	0.010

*Stage*							
IA	646 (17.0)	—	—	—	—	—	—
IB	674 (17.8)	1.37	1.18–1.60	<0.001	1.29	1.11–1.50	<0.001
IIA	95 (2.6)	1.64	1.23–2.27	<0.001	1.07	0.79–1.45	0.001
IIB	206 (5.5)	1.73	1.42–2.12	<0.001	1.26	1.01–1.56	0.660
IIIA	476 (12.5)	2.10	1.80–2.45	<0.001	1.21	1.00–1.47	0.042
IIIB	702 (18.5)	2.04	1.78–2.36	<0.001	1.49	1.27–1.75	0.056
IV	992 (26.1)	3.60	3.15–4.11	<0.001	2.36	2.02–2.77	<0.001

*LN metastasis*							
No/unknown	1990 (52.4)	—	—	—	—	—	—
Yes	1801 (47.6)	2.02	1.86–2.18	<0.001	1.51	1.34–1.70	<0.001

*Surgery*							
Yes-yes	1285 (34.0)	—	—	—	—	—	—
Yes-no	1514 (39.9)	2.33	2.11–2.56	<0.001	1.81	1.61–2.02	<0.001
No-yes	185 (4.8)	1.36	1.12–1.67	<0.001	0.94	0.76–1.16	0.536
No-no	807 (21.3)	2.87	2.57–3.22	<0.001	1.92	1.68–2.19	<0.001

*Radiation*							
Yes-yes	531 (14.1)	1.35	1.19–1.52				
Yes-no	516 (13.6)	1.68	1.50–1.89				
No-yes	970 (25.6)	1.19	1.08–1.31				
No-no	1774 (46.7)	—	—				

*Chemotherapy*							
Yes-yes	520 (13.7)	1.48	1.32–1.65				
Yes-no	790 (20.8)	1.00	0.89–1.11				
No-yes	637 (16.9)	1.49	1.34–1.66				
No-no	1844 (48.6)	—	—				

## Data Availability

The data supporting the results reported in this article can be available by contacting the corresponding author.

## Abbreviations

FPLC: First primary lung cancer
SPLC: Second primary lung cancer
DPLC: Dual primary lung cancer
MPLC: Multiple primary lung cancer
OS: Overall survival
HR: Hazards ratio
ROC: Receiver-operating characteristic
AUC: Area under the curve
C-index: Concordance index
PSM: Propensity score matching
SEER: Surveillance, Epidemiology, and End Results.

## Supplementary Materials

Supplementary Materials Figure S1: flow chart detailing the selection of the patients in this study. MP-SIR, multiple primary-standard incidence rate; ICD, International Classification of Diseases for Oncology; AJCC, American Joint Committee on Cancer. Figure S2: ROC curves. ROC curve analyses were generated to evaluate the predictive value of the established nomogram by the AUC. A and B came from the modeling (3-year and 5-year OS, resp.). C and D came from the validation group (3-year and 5-year OS, resp.). Figure S3: distribution of propensity scores. The treatment units represent surgery group, and the control units represent no surgery group. Each circle represents one patient. The size of the circles for matched patients is proportional to the distance obtained by the propensity score matching procedure. Table S1: C-index and AUC for the established nomogram to predict 3-year and 5-year overall survival. Table S2: characteristics of patients before and after matching included in the study.
