# Supplementary material for: Prognostic Factors Analysis and Nomogram Construction of Dual Primary Lung Cancer: A Population Study
Source: Biomed Res Int. 2020 Feb 19;2020:7206591. doi: 10.1155/2020/7206591 (PMC7049836; doi:10.1155/2020/7206591)
Supplement: Supplementary Materials — Figure S1: flow chart detailing the selection of the patients in this study. MP-SIR, multiple primary-standard incidence rate; ICD, International Classification of Diseases for Oncology; AJCC, American Joint Committee on Cancer. Figure S2: ROC curves. ROC curve analyses were generated to evaluate the predictive value of the established nomogram by the AUC. A and B came from the modeling (3-year and 5-year OS, resp.). C and D came from the validation group (3-year and 5-year OS, resp.). Figure S3: distribution of propensity scores. The treatment units represent surgery group, and the control units represent no surgery group. Each circle represents one patient. The size of the circles for matched patients is proportional to the distance obtained by the propensity score matching procedure. Table S1: C-index and AUC for the established nomogram to predict 3-year and 5-year overall survival. Table S2: characteristics of patients before and after matching included in the study. [file 7206591.f1.zip › Table S1.docx]

**Table 3**: **C-index and AUC for the established nomogram to predict 3-year and 5-year overall survival.**

| \| **Indicators** \| \| --- \| \| | \| **Modeling group** \| \| --- \| | | \| **Verification group** \| \| --- \| | |
| --- | --- | --- | --- | --- | --- | --- | --- |
|  | **3-year OS** | **5-year OS** | **3-year OS** | **5-year OS** |
| **AUC** | 0.72 | 0.73 | 0.72 | 0.74 |
| **C-Index (95% CI)** | 0.70 ( 0.69 to 0.71 ) | | 0.70 ( 0.68 to 0.72) | |

Abbreviations: OS, Overall Survival; AUC, Area Under Curve; C-index, index of Concordance; CI, Confidence Interval.
